# Supplementary material for: An intranasally delivered peptide drug ameliorates cognitive decline in Alzheimer transgenic mice
Source: EMBO Mol Med. 2017 Mar 29;9(5):703–15. doi: 10.15252/emmm.201606666 (PMC5412883; doi:10.15252/emmm.201606666)
Supplement: Supplementary file 1 — Appendix [file EMMM-9-703-s001.pdf]

An intranasally delivered peptide drug ameliorates the cognitive decline in Alzheimer transgenic mice

Yu-Sung Cheng[1†], Zih-ten Chen[2†],Tai-Yan Liao[2], Chen Lin[2], Howard C.-H. Shen[2], Ya-Han Wang[2,5], Chi-Wei Chang[3], Ren-Shyan Liu[3,4] Rita P.-Y. Chen[2,5*], Pang-hsien Tu[1*]

Appendix

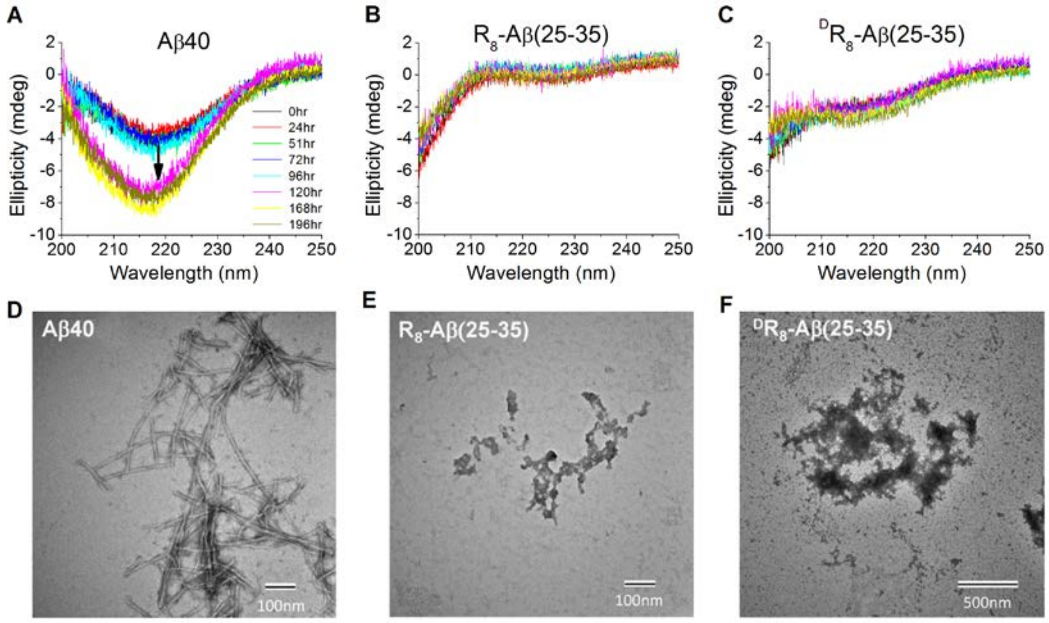

Appendix Figure S1. CD spectra and TEM images of Aβ_{40}, R_8-Aβ(25-35), and $^{D}R_8$-Aβ(25-35). The peptides were dissolved in 20 mM sodium phosphate buffer with 150 mM KCl and incubated at 25°C. Peptide concentration is 30 μM. (A-C) The CD spectra were recorded at the indicated incubation time shown in (A). (D-F) The TEM images were taken after incubated for 168 hours. This batch of Aβ_{40} formed nucleus very fast and β-sheet signal (negative ellipticity at 218 nm) can be seen in the beginning of incubation. The CD spectra were not smoothed.

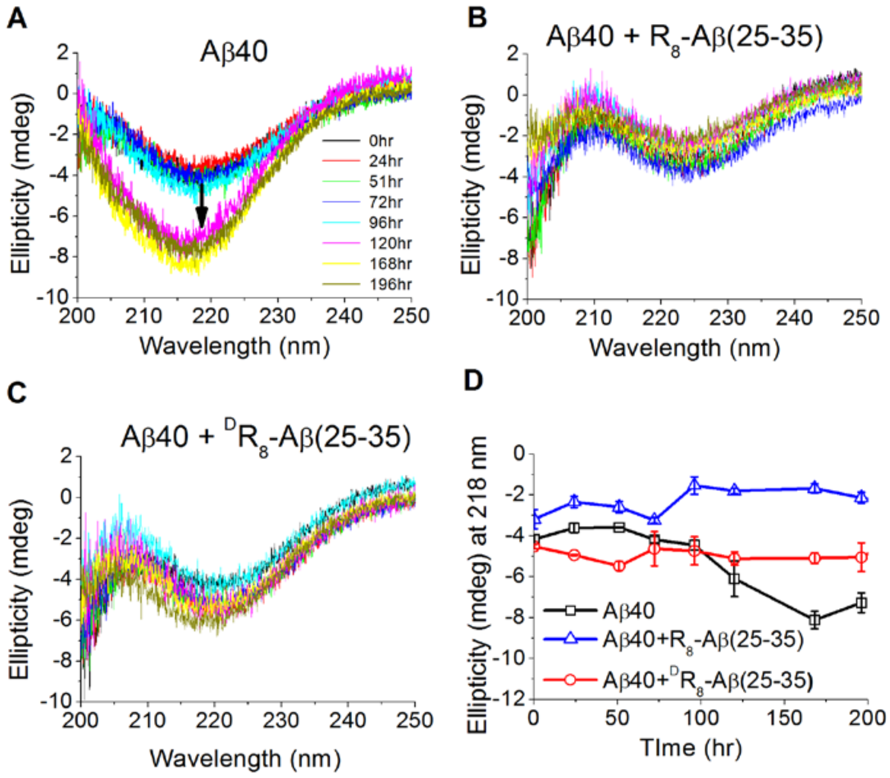

Appendix Figure S2. Effect of the designed bipartite peptides on inhibition of Aβ_{40} fibrillization. The peptides were dissolved in 20 mM sodium phosphate buffer with 150 mM KCl and incubated at 25°C. Peptide concentration is 30 μM for each peptide. The CD spectra of Aβ_{40} (A), the Aβ_{40}/R_8-Aβ(25-35) mixture (1:1) (B), and the Aβ_{40}/$^{D}R_8$-Aβ(25-35) mixture (1:1) (C) were recorded at the indicated incubation times shown in (A). The spectrum change is pointed out by an arrow in (A), indicating the formation of cross-β structure in the amyloid fibrils. (D) The time course of amyloidogenesis of Aβ_{40} with and without the designed peptide inhibitors. The CD spectra were not smoothed. (A) is the same as Fig. S1A.

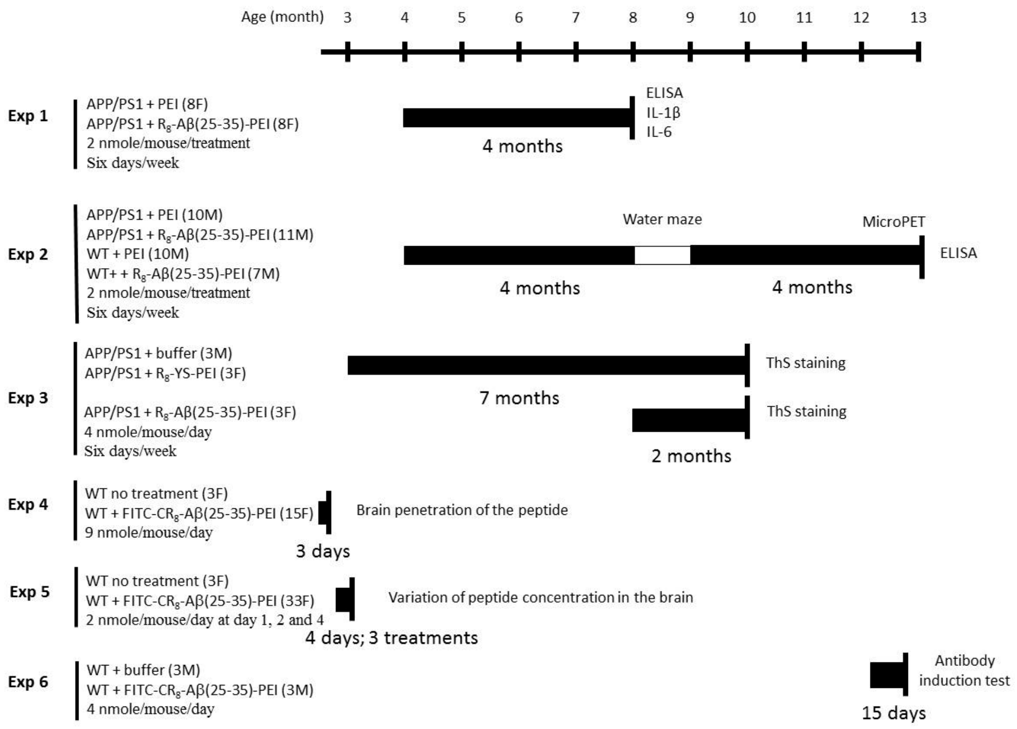

Appendix Figure S3. Designs of animal experiments. The mice were kept in individually ventilated cages (IVC) on a 12:12 h light:dark cycle. The mice had access to food and water ad libitum. *APP/PS1* (B6C3-Tg(APPswe,PSEN1dE9)85Dbo/Mmjax) transgenic mice were purchased from Jackson Laboratories (Bar Harbor, Maine, USA) and bred and genotyped as described on the Jackson website. Wildtype C57BL/6JNarl mice were purchased from National Laboratory Animal Center (Taiwan). No specific blinding procedure was taken.

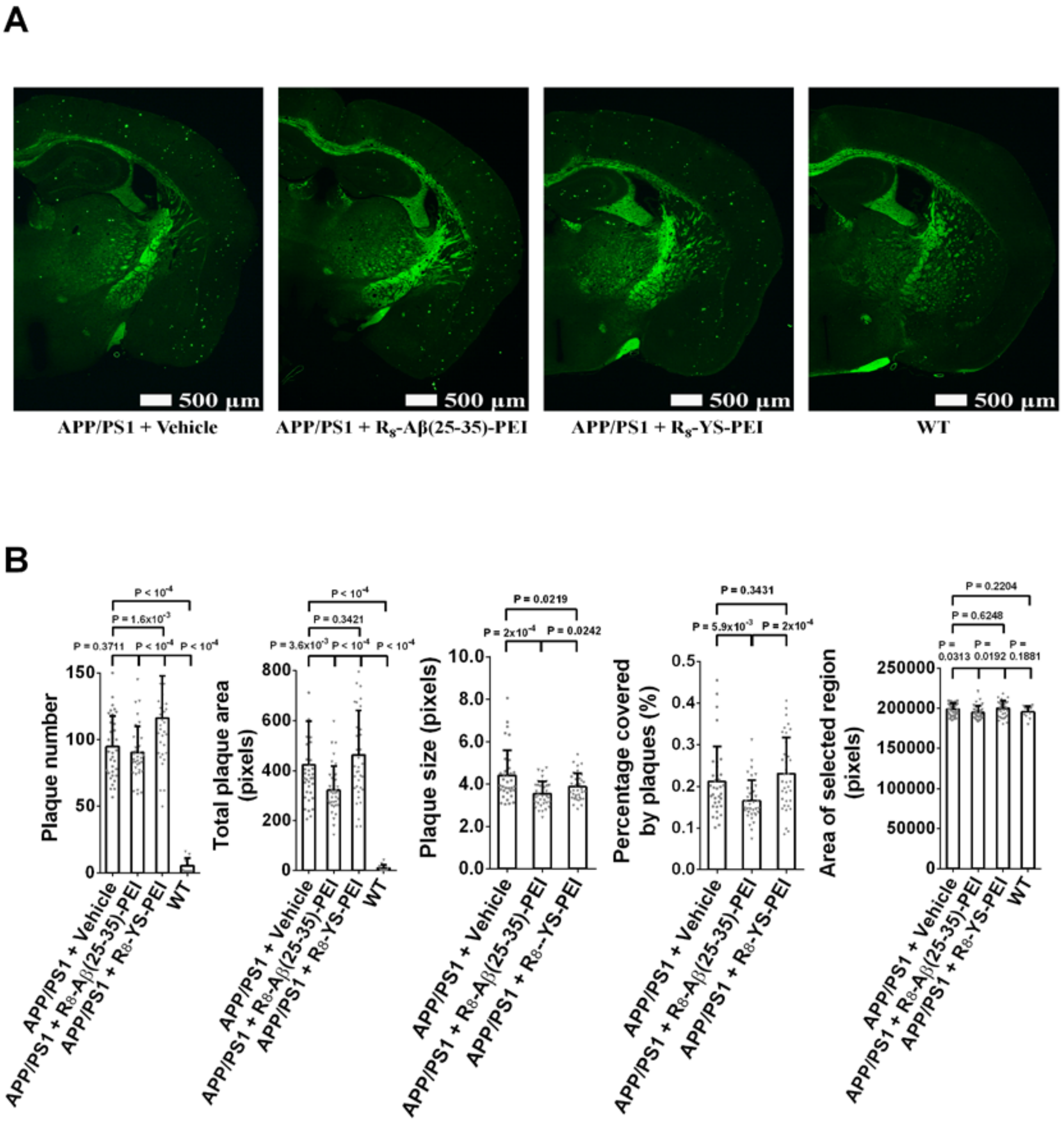

Appendix Figure S4. Effect of R$_8$-Aβ(25-35)-PEI on reducing amyloid plaque accumulation. R$_8$-Aβ25-35-PEI and R$_8$-YS-PEI was dissolved in 100 mM NaH_2PO_4/138 mM KCl (pH 5) to a concentration of 800 μM. The APP/PS1 mice were treated intranasally with the buffer or R$_8$-YS-PEI from the age of 3 to 10 months and R$_8$-Aβ(25-35)-PEI from the age of 8 to 10 months (2.5 μL was given daily to each nostril, six days per week). Three mice in each group. (A) Representative ThS-staining image of each group compared with non-transgenic littermates (WT). (B) Statistical analysis of amyloid plaque accumulation per brain section. Twelve sections per mouse were stained and analyzed. Data were expressed in mean ± SD. The statistics were done by Student's *t*-test.

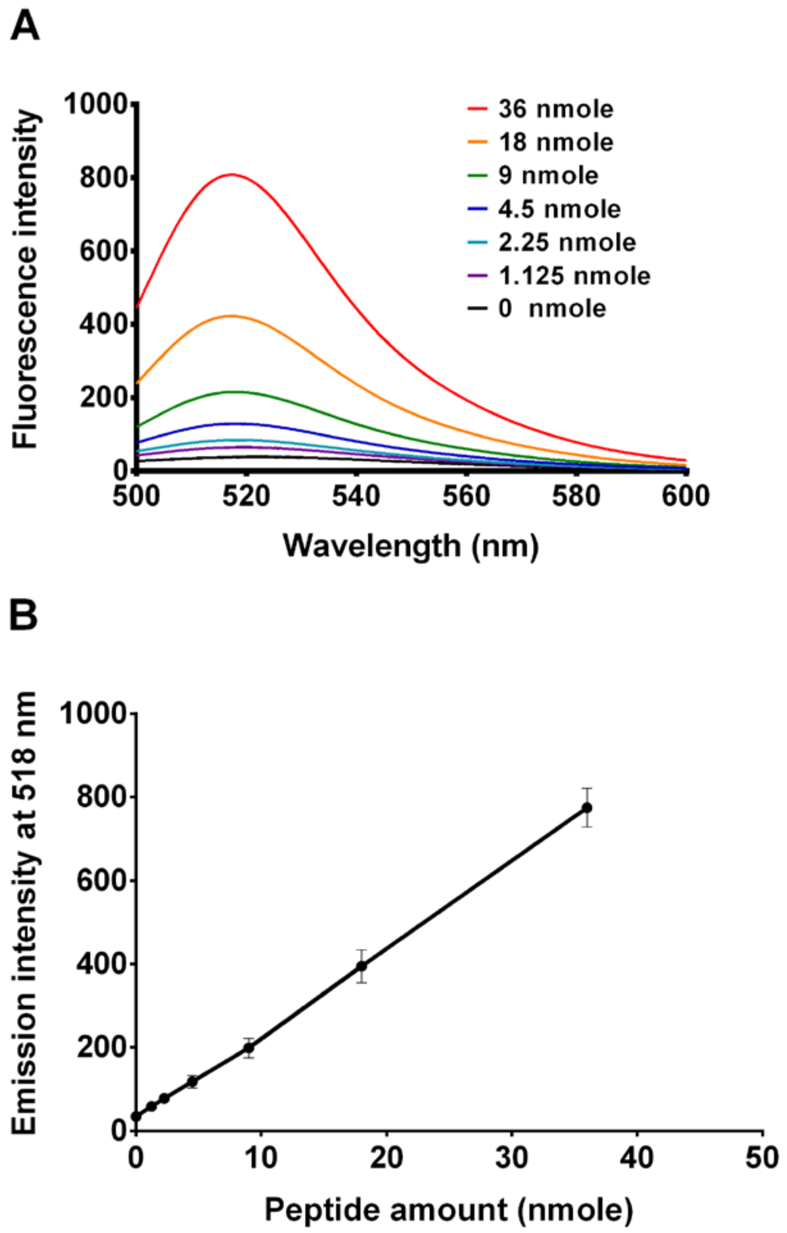

Appendix Figure S5. Calibration of FITC-(Ahx)-CR$_8$-Aβ(25-35)-PEI amount in the brain. (A) Fluorescence spectra of the filtrate of mouse brain homogenate containing different amount of peptide. (B) The plot of fluorescence intensity at 518 nm versus peptide amount. Data were expressed in mean ± SD (two independent brain samples). The peptide amount is the total peptide amount in 1.5 mL of filtrate.

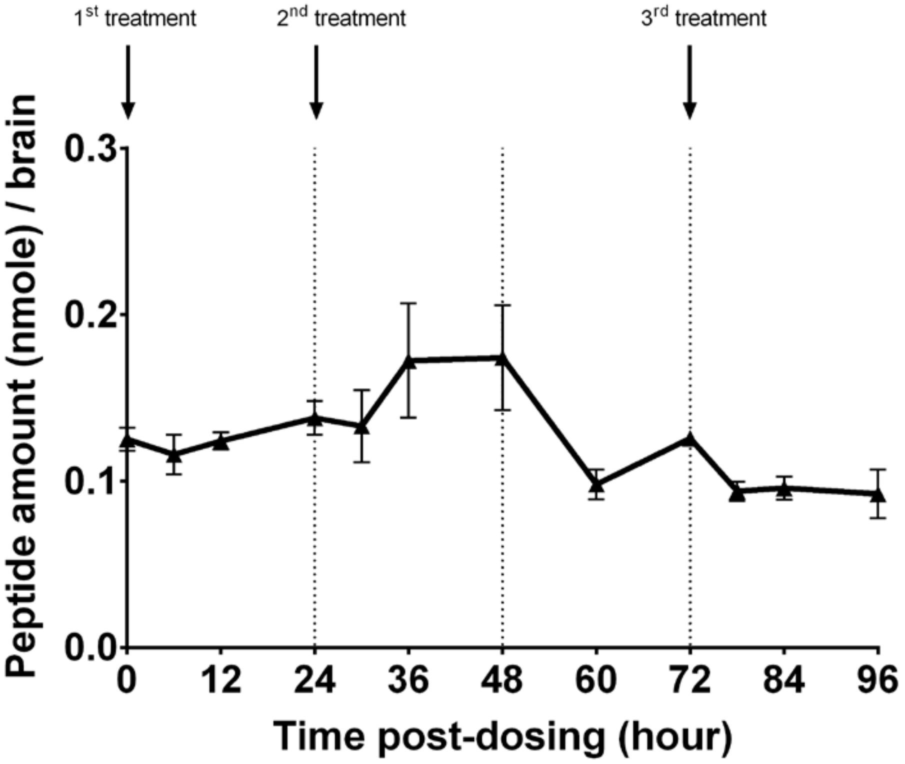

Appendix Figure S6. Concentration variation of FITC-(Ahx)-CR_8-Aβ(25-35)-PEI in the mouse brain during consecutive intranasal treatment. Twelve-week old female wildtype C57BL/6JNarl mice were treated intranasally with FITC-(Ahx)-CR_8-Aβ(25-35)-PEI (2 nmole/mouse/treatment) at 0 h, 24 h, 72 h. At the indicated times three mice were sacrificed and their brains were perfused. The brains were collected and homogenized by sonication on ice as described in the method section. The homogenate was centrifuged at 21000 g for 20 min. After centrifugation, the supernatant was passed through a microconcentrator (100K cut-off, Pall Gelman, USA). Each filtrate was adjusted with the homogenization buffer to make a final volume of 1.0 mL and then their fluorescence emission spectra (500-600 nm) were recorded on a fluorescence spectrophotometer (FP-750, Jasco, Japan) with excitation at 446 nm. The pathlength was 1 cm and slit width was 10 nm for both excitation and emission. The amounts of FITC-(Ahx)-CR_8-Aβ(25-35)-PEI per brain at 0, 6, 12, 24, 30, 36, 48, 60, 72, 78, 84, 96 h were ploted. Data were expressed in mean ± SD (n=3).

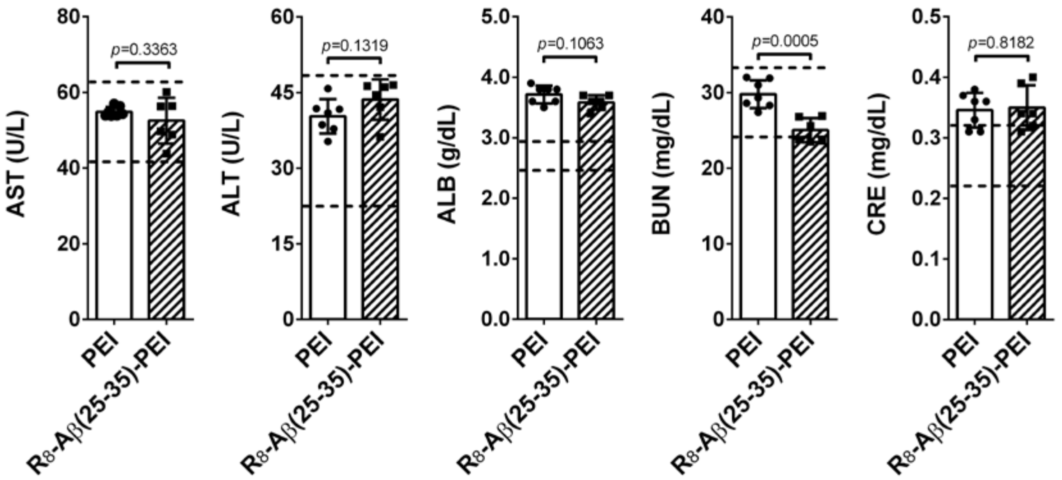

Appendix Figure S7. Liver and kidney toxicity test for the APP/PS1 mice after the R_8-Aβ(25-35)-PEI treatment from the age of 4 months to 13 months (with a one-month break in the middle). Blood samples of PEI or R_8-Aβ(25-35)-PEI treated APP/PS1 mice were collected. Serum aspartate transaminase (AST), alanine transaminase (ALT), albumin (ALB), blood urea nitrogen (BUN) and creatinine (CRE) levels were then analyzed by Fuji Dri-chem 4000i Analyzer (Taiwan Mouse Clinic). All results were expressed in mean ± SD (n=7 for PEI treatment; n=6 for R_8-Aβ(25-35)-PEI treatment). The statistics were done by Student's *t* test. The range of AST, ALT, ALB, BUN, CRE levels obtained from 10-wk-old ICR mice (n=10) are shown in dash line.

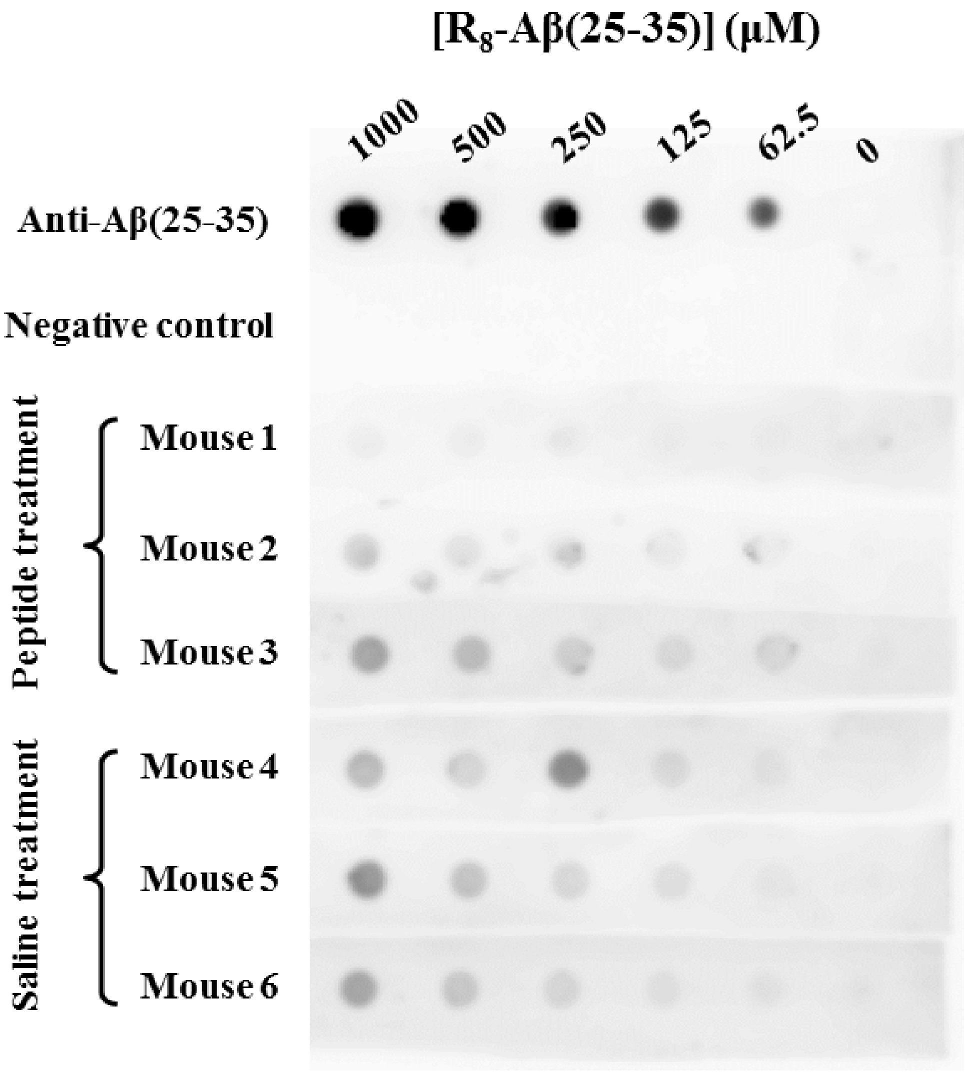

Appendix Figure S8. Dot plot of R_8-Aβ(25-35). The peptide concentration was indicated above. The row 1 is immubloted by anti- Aβ(25-35) antibody. The row 2 is negative control. The row 3-8 was immublotted by the sera collected from the mice with peptide or saline treatment. The results indicated that no antibody was induced against the R_8-Aβ(25-35) peptide after intranasal peptide treatment.

Supplemental methods

Dot blot assay

FITC-R_8-Aβ(25-35)-PEI peptide was dissolved in 100 mM NaH_2PO_4/138 mM KCl (pH 5) to 800 μM. Six one-year old male non-transgenic littermates were treated intranasally with FITC-(Ahx)-CR_8-Aβ(25-35)-PEI peptide or buffer (5 μL/mouse/d) for 15 days (n=3 per group). Two hours after the last treatment, blood was collected from the facial vein of these mice. The blood was centrifuged and the serum was analyzed by dot blot to identify whether any antibody against this peptide was induced. Eight rows of R_8-Aβ(25-35) were prepared on a nitrocellulose membrane (0.45 μm, GE Healthcare, UK). Each row was loaded with 50 μL of R_8-Aβ(25-35) (1000, 500, 250, 125, and 62.5 μM). The membrane was blocked in the blocking-solution containing 5% skimmed milk in Tris-buffered saline for 1 h. The membrane was then cut into 8 strips. As a positive control, strip 1 was incubated with polyclonal anti-β-Amyloid (25-35) antibody (A00687-40, 1:1000 diluted, GenScript, USA), washed, and incubated with a horseradish peroxidase (HRP)-conjugated secondary antibodies at room temperature for 1 h (goat anti-rabbit IgG antibody AP132P, Millipore, USA). Strip 2 was directly incubated with a horseradish peroxidase (HRP)-conjugated secondary antibody (goat anti-mouse IgG antibody HAF007, R&D system, USA) at room temperature for 1 h as negative control. For testing mouse sera, strips 3-8 were incubated with the serum (1:100 diluted) taken from peptide- or buffer-treated mice individually for 1h at room temperature. After washing, strips 3-8 were incubated with a horseradish peroxidase (HRP)-conjugated secondary antibody (goat anti-mouse IgG antibody HAF007, R&D systems, USA) at room temperature for 1 h. Signals were visualized using a luminescent image analyzer (LAS4000, GE Healthcare, UK).
